# Periodontal conditions in a 65-year-old population and prevalence of periodontitis according to three different bone level thresholds

**DOI:** 10.1186/s12903-022-02276-1

**Published:** 2022-06-21

**Authors:** Anne Thea Tveit Sødal, Lene Hystad Hove, My Tien Diep, Rasa Skudutyte-Rysstad, Odd Carsten Koldsland

**Affiliations:** 1 Department of Cariology and Gerodontology, Faculty of Dentistry, University of Oslo, Blindern, P.O. Box 1109, 0317 Oslo, Norway; 2 Department of Periodontology, Faculty of Dentistry, University of Oslo, Oslo, Norway

**Keywords:** Alveolar bone loss, Classification, Periodontal diseases, Periodontitis, Prevalence

## Abstract

**Aims:**

The aims of this cross-sectional study were to describe the prevalence and severity of periodontal disease in a 65-year-old population in Oslo, Norway, and to investigate to what extent the radiographic bone level threshold for periodontitis case definition influences the prevalence.

**Materials and methods:**

A random sample of 454 subjects underwent a clinical and radiographic examination and answered a questionnaire regarding general health, medications, and smoking habits. Clinical periodontal parameters (periodontal pocket depths, bleeding on probing, mobility, and furcation involvement) and radiographic bone loss were used to identify periodontitis cases and to assess periodontal stage and grade.

**Results:**

Of the 454 participants, 52.6% were defined as “periodontitis cases”. Of the total study population “unstable cases of recurrent periodontitis” were present in 38.1%, 16.5% of the participants were assigned to stage II, 32.8% to stage III, and 3.3% to stage IV. When lowering the radiographic bone loss cutoff from > 3 mm to > 2 mm or > 1 mm the prevalence of periodontitis increased to 91.9% and 99.6%.

**Conclusions:**

Periodontitis was common among 65 year-olds living in Oslo, and in the majority of those with periodontitis, the disease was recurrent and unstable. This study also shows that the choice of bone loss cutoff for defining a periodontitis case affects the prevalence estimates to a large extent. In addition, this study addresses weaknesses in the use of the consensus report of the 2017 World Workshop on the Classification of Periodontal and Peri-implant Diseases and Conditions for epidemiologic studies in its current form.

## Introduction

Periodontitis is an inflammatory disease affecting the supportive tissues of the teeth, and may lead to tooth loss if left untreated [1–3]. In Norway, patients' expenses for periodontal treatment have partly been subsidized by the public health insurance system for two decades [4]. Despite this public financial support and readily accessible dental health services, recent studies from different parts of Norway have shown a periodontitis prevalence ranging from 49 to 72% [5–7] with higher prevalence in older age groups. However, age-specific prevalence in a large sample of young elderly have not been reported. In a national population study from the U.S. the prevalence of periodontitis was estimated to be 71.5% among adults 65–74 years of age [8]. Due to a higher proportion of the elderly retaining their natural teeth [9–11], an increased need for periodontal treatment in the population may occur. Therefore, updated prevalence data in the young elderly will be of great significance for planning future dental health services for the growing population of elderly. In addition, periodontitis has been shown to affect oral health-related quality of life [12]. Periodontitis may also increase the risk of complications of systemic diseases [1] and has shown associations to systemic diseases that are highly prevalent with increasing age [13, 14]. A high prevalence of periodontal disease among older adults, along with the increasing proportion of elderly in the world population [15] may therefore have an impact on the need for health care services in the years to come.

There is a large variation in definitions and classifications of periodontitis used in previous studies. This might partly explain the differences in reported prevalence data, and makes comparison of results from different studies challenging as previously described [16, 17]. The case definition developed by the Centers for Disease Control (CDC) and Prevention and the American Academy of Periodontology (AAP) [18] has been widely used in epidemiological studies [16]. However, new definitions and diagnostic criteria were presented in the 2017 World Workshop on the Classification of Periodontal and Peri-implant Diseases and Conditions [19] organized by AAP and the European Federation of Periodontology (EFP). Ke et al. found a large variation in the periodontitis prevalence estimates when comparing the use of the consensus report from 2017 and other periodontal classifications [20]. However, only CAL cutoffs for stages were used, and further investigation on the epidemiological utility of the consensus report containing more of the parameters required to predict the severity and prognosis of periodontitis was requested.

The aims of the present study were therefore to describe the prevalence and severity of periodontal disease in a 65-year-old population in Oslo, Norway based on the 2017 World Workshop on the Classification of Periodontal and Peri-implant Diseases and Conditions, and to investigate to what extent the choice of radiographic bone level cutoffs for periodontitis case definition influences the disease prevalence estimates.

## Material and methods

### Participants

In this cross-sectional study, oral health in a 65-year-old population in Norway was investigated. A random sample was drawn from the Norwegian Population Register (retrieved from the Norwegian Tax Administration) and invitation letters were sent out. Inclusion criteria were “born in 1954” and “resident in Oslo”. All individuals who received the letter and were reachable by phone were contacted and given the opportunity to participate in the study. The recruitment procedure has been described in detail in a previous publication [21]. The study was approved by the Norwegian Regional Committee for Medical and Health Research Ethics (REK 2018/1383) and performed in compliance with the tenets of the Declaration of Helsinki. A written informed consent was signed by each participant prior to the clinical examination.

### Questionnaire

All participants answered a semi-structured questionnaire prior to the clinical examination using the Nettskjema software (University of Oslo, Norway). The questionnaire contained items regarding general diseases, medication use, and smoking habits. Self-reported diabetes type 1 and 2 were assessed by yes/no questions. Smoking habits were assessed by the three response alternatives: “never smoker”, “former smoker”, and “current smoker”. “Current smoker” was defined as an individual who smoked at least one cigarette daily. Current smokers also reported the number of cigarettes daily consumed. The participants’ country of birth was dichotomized into ‘western’ (Nordic countries, Western Europe, North America, and Australia) and ‘non-western’ (the rest of the world). The level of education was dichotomized into ‘higher education’ (university/college education) and ‘basic education’ (high school, elementary school, or lower).

### Clinical periodontal examination

Two trained, calibrated dentists (ATTS and MTD) performed all clinical examinations at the Research Clinic at the Faculty of Dentistry, University of Oslo from February to December 2019. Periodontal probing depths (PPD), bleeding on probing (BoP) and suppuration were measured on six sites per tooth using LM 52B XSI Perio Probe (LM-Dental™, Planmeca Group, Helsinki, Finland). PPD was recorded to the nearest mm, rounded down. Tooth mobility was recorded as grade 1, 2, or 3 as described by Nyman et al. [22]. Furcation involvement was measured on molars using Nabers Q2N probe (Hu-Friedy, Chicago, USA) and recorded as grade 0, I, II, or III [23]. Third molars were not included in the clinical registrations.

The examiners were trained by a specialist in periodontology (OCK) prior to inter-rater calibration and clinical examination. Inter-rater reliability of the clinical examiners was estimated from pocket depth registrations from seven participants, a total of 336 values per examiner. The intra-class correlation coefficient (ICC) (95% CI) was 0.82 (0.78–0.86).

### Radiographic examination

Orthopantograms (OPG) and horizontal bitewings (BW) were used to assess radiographic bone loss (RBL). The OPGs were obtained using a panoramic imaging unit (ProMax X-ray Dimax 3 and Planmeca ProOne, Planmeca Oy, Helsinki). Two BWs per participant were obtained using an intraoral imaging unit (MINIRAY, SOREDEX, PaloDEx Group Oy, Tuusula, Finland) with a rectangular collimator (length 30.5 cm). Percentage bone loss was calculated for each tooth by dividing the distance from cemento-enamel junction to alveolar crest by the distance from the cemento-enamel junction to apex measured on OPGs. In addition, detectable interproximal bone loss was recorded as the distance in millimeters from the cemento-enamel junction to the alveolar bone crest on the two most severely affected non-adjacent sites on BW.

Radiographic registrations were performed by one calibrated dentist (ATTS) in a room with adapted ambient light and by applying measuring instruments in the ImageJ software (ImageJ 1.52a, National Institutes of Health, USA). Calibrations were performed on OPGs from 50 participants. A specialist in periodontology (OCK) and a trained dentist (ATTS) separately evaluated 50 radiographs. The results were evaluated and for the inter-examiner calibration the ICC (95% CI) was 0.79 (0.66–0.86) for % bone loss and the weighted Cohen’s kappa (95% CI) was 0.72 (0.66–0.78) for stage. Intra-examiner calibration was performed (ATTS) on 25 radiographs. For the intra-examiner radiographic registration the ICC (95% CI) was 0.88 (0.86–0.90) for % bone loss and the weighted Cohen’s kappa (95% CI) was 0.90 (0.82–0.98) for stage.

### Definitions

The results from the clinical and radiographic examination in the present study were classified based on the 2017 World Workshop on the Classification of Periodontal and Peri-implant Diseases and Conditions [19, 24, 25]. A periodontitis case was defined as an individual with detectible RBL (distance from the cemento-enamel junction (CEJ) to the alveolar bone crest (AC) measured on BW radiographs) exceeding 3 mm on ≥ 2 non-adjacent teeth. For further classification of disease activity, the following definitions based on Sanz et al. [26] were used:

- Periodontitis case, stable case of periodontal health: PPD ≤ 4 mm, BoP < 10%, no PPD ≥ 4 mm with BoP
- Periodontitis case, some gingival inflammation: PPD ≤ 4 mm, BoP ≥ 10%, no PPD = 4 mm with BoP
- Periodontitis case, unstable case of recurrent periodontitis: PPD ≥ 5 mm, or PPD ≥ 4 mm with BoP

Percentage of radiographic bone loss measured on each tooth was used for evaluating the severity of periodontitis according to Papapanou et al. [19]: < 15%; stage I, 15–33%; stage II, extending to mid-third of root and beyond; stage III. In addition, PPD, furcation involvement, and tooth loss due to periodontitis were investigated as complexity factors and used for further staging of the periodontitis cases as described by Papapanou et al. [19]. Vertical bone loss was registered if the radiographic defect was ≥ 3 mm deep and ≤ 3 mm wide [6]. Teeth were registered as lost due to periodontitis based on clinical judgment if this was considered as the most likely cause for tooth loss, that is, cases where no other reasons for tooth loss such as caries, endodontic lesions, root canal treatments seemed reasonable, and the general bone level in remaining dentition suggested periodontal tooth loss. In cases of doubt, tooth loss was not assigned to periodontitis. For each stage group the extent was described as localized (< 30% of teeth involved) or generalized (≥ 30% of teeth involved).

For grading of periodontitis cases, percent bone loss at the most affected tooth divided by age was calculated and categorized as grade A (< 0.25), B (0.25–1.0), or C (> 1.0). In addition, smoking and diabetes were treated as grade modifiers. The grade modifiers could only shift the grade, based on radiographic bone loss, to a higher level. Smokers who smoked < 10 cigarettes daily were categorized as grade B and individuals who smoked ≥ 10 cigarettes daily were categorized as grade C. In lack of hemoglobin A1c measurements, diabetes could only shift the grade from A to B and not from B to C.

Staging and grading were only performed on periodontitis cases. For non-periodontitis cases, the following definitions based on Chapple et al. [24] were used:

- Cases with periodontal health: No detectable bone loss and BoP < 10%
- Gingivitis cases: No detectable bone loss and BoP ≥ 10%

In cases with no detectable bone loss, PPD > 3 mm and BoP < 10%, pocket depths were assigned to anatomical causes, and the participants were defined as cases with periodontal health. In cases with PPD > 3 mm, no detectable bone loss on radiographs, and BoP ≥ 10%, pocket depths were considered as pseudo pockets and the participants were assigned to the gingivitis group. Gingivitis was defined as localized when BoP was present in < 10% of sites and generalized when BoP was present in ≥ 10% of sites.

In addition to the 3 mm interproximal RBL cutoff used for identifying periodontitis cases, the difference in prevalence, stages, and grades when using RBL > 1 mm and RBL > 2 mm cutoffs were investigated.

### Statistical analyses

Clinical and radiographic registrations were collected in The Oral Data Collector sheet specifically designed for data entry in this study, developed in Microsoft Excel 2016 (Microsoft Corporation, Redmond, Washington, US), and imported into STATA (Stata version 16.1; College Station, TX, USA) for statistical analysis. Data were stored in Service for Sensitive Data (TSD facilities, UiO). Participants with ≤ 1 remaining tooth were excluded from the analyses. The results from the descriptive analyses are presented as percentage distributions or mean and standard deviation (SD).

## Results

### Participants

**Fig. 1:**
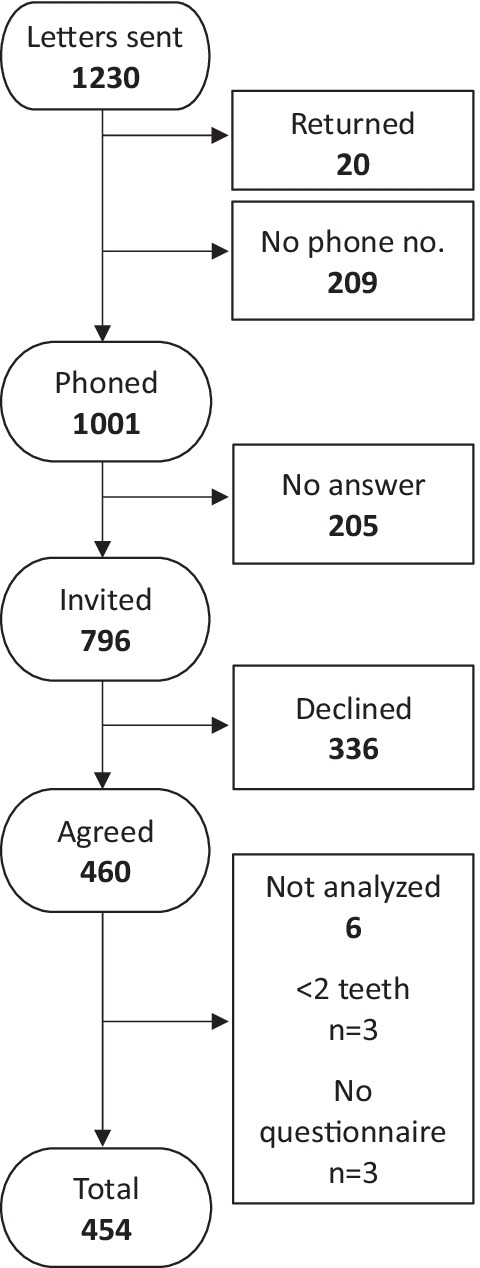
Study participants

The recruitment process is presented in Fig. 1. Distribution with respect to participants' characteristics is presented in Table 1.

**Table 1:** Background characteristics of participants

Participants' characteristics	n (%)
	(N = 454)
*Gender*	
Men	233 (51.3)
Women	221 (48.7)
*Country of birth*	
Western	413 (91.0)
Non-western	41 (9.0)
*Education level*	
Higher education	304 (67.0)
Basic education	150 (33.0)
*Smoking*	
Never-smoker	197 (43.4)
Former-smoker	210 (46.3)
Smoker < 10/day	18 (4.0)
Smoker ≥ 10/day	29 (6.4)
*Diabetes*	
No	420 (92.5)
Type 1	4 (0.9)
Type 2	30 (6.6)

### Clinical periodontal parameters

In the present population, the average number of teeth present was 25.6 (SD = 3.4), ranging from 4 to 28 remaining teeth. Distributions of participants with respect to BoP and PPD are presented in Table 2.

**Table 2:** Distribution of participants with respect to proportion of sites with bleeding on probing (BoP) and the most severe periodontal probing depth (PPD) (N = 454)

	n (%)
*% of sites with BoP*	
< 10%	341 (75.1)
10–30%	98 (21.6)
> 30%	15 (3.3)
*Maximum PPD*	
≤ 3 mm	123 (27.1)
4–5 mm	210 (46.3)
6–8 mm	101 (22.2)
9–10 mm	14 (3.1)
≥ 11 mm	6 (3.1)

### Periodontitis prevalence, periodontal health, and gingivitis

**Fig. 2:**
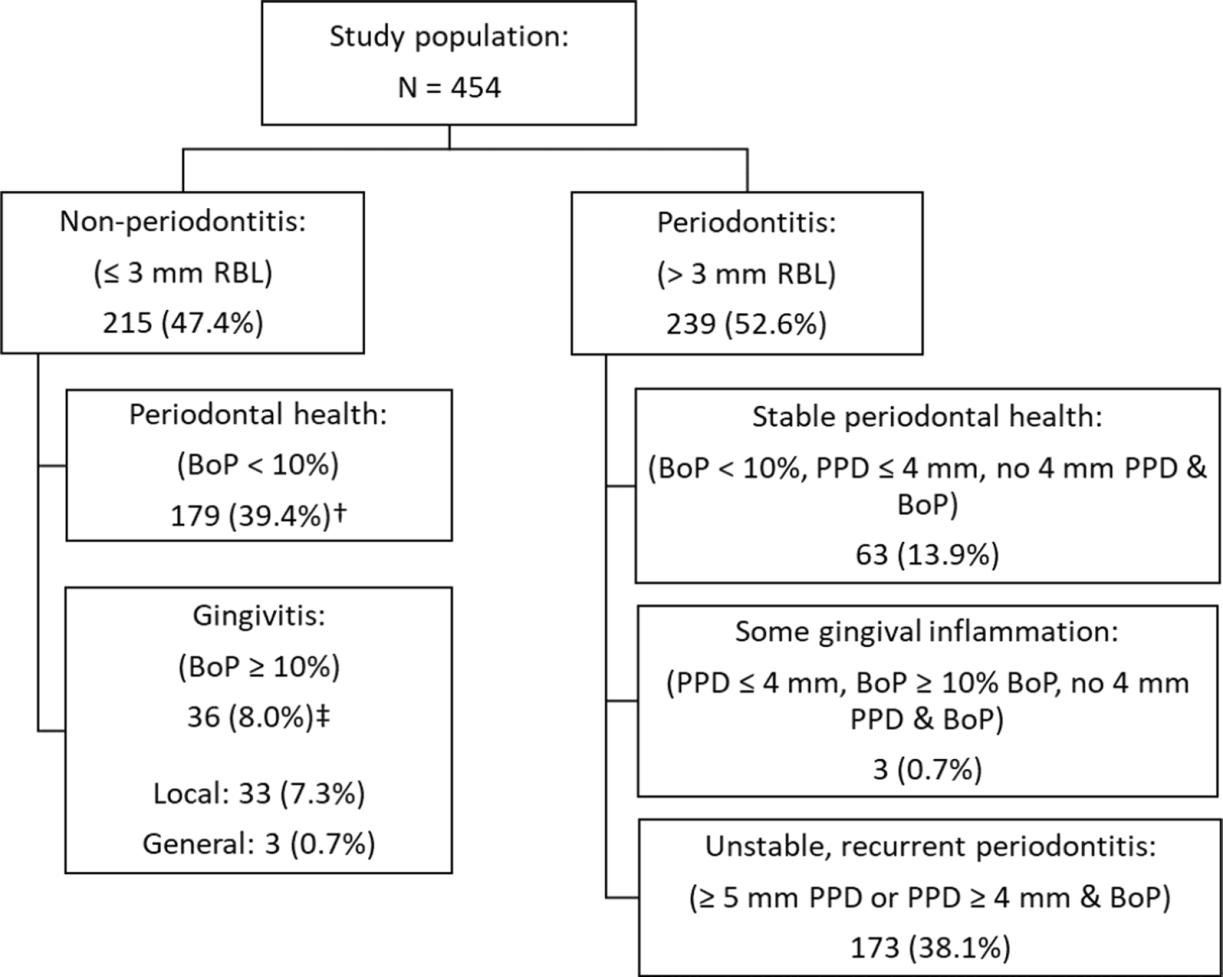
Distribution of participants with respect to periodontal health, gingivitis and periodontitis presented as n (%). ^†^Participants with PPD > 3 mm regarded as anatomical pockets (n = 100). ^‡^Participants with PPD > 3 mm regarded as pseudo pockets (n = 29)

Figure 2 shows the prevalence of periodontitis (52.6%) and non-periodontitis cases (47.4%) according to the 3 mm interproximal RBL cutoff. In addition, the distribution of participants with respect to periodontal health (39.4%), gingivitis (8%), and periodontal disease activity are shown in Fig. 2.

### Staging

In the study population, staging based only on RBL, resulted in 125 (27.5%) periodontitis cases assigned to stage II and 114 (25.1%) to stage III. In twenty-seven cases (5.9%) tooth loss was assigned to periodontitis, of which 12 participants (2.6%) had lost 1–4 teeth and 15 participants (3.3%) had lost ≥ 5 teeth. Of the participants classified as periodontitis cases, 96 individuals had PPD of ≤ 5 mm and 143 individuals had PPD ≥ 6 mm. Vertical bone loss ≥ 3 mm was detected in 18 periodontitis cases, while furcation involvement grade 2 or 3 was identified in 81 periodontitis cases. Distribution of periodontitis cases with respect to stage, when complexity factors are taken into account, and also disease activity are shown in Table 3.

**Table 3:** Distribution of periodontitis cases with respect to stage and disease activity, extent, and grade (N = 454)

	Non-periodontitis cases	Stage I	Stage II	Stage III	Stage IV
	n (%)	n (%)	n (%)	n (%)	n (%)
Total	215 (47.4)	0 (0)	75 (16.5)	149 (32.8)	15 (3.3)
*Disease activity*					
Periodontitis case with stable periodontal health	–	0 (0)	37 (8.1)	26 (5.7)	0 (0)
Periodontitis with some gingival inflammation	–	0 (0)	3 (0.7)	0 (0)	0 (0)
Unstable case of recurrent periodontitis	–	0 (0)	35 (7.7)	123 (27.1)	15 (3.3)
*Extent of periodontal stage*					
Localized	–	0 (0)	5 (1.1)	138 (30.4)	0 (0)
Generalized	–	0 (0)	70 (15.4)	11 (2.4)	15 (3.3)
*Grade*					
A	–	0 (0)	0 (0)	0 (0)	0 (0)
B	–	0 (0)	73 (16.1)	124 (27.3)	6 (1.3)
C	–	0 (0)	2 (0.4)	25 (5.5)	9 (2.0)

### Extent

In the total study population, 21.1% showed generalized and 31.5% showed localized disease based on radiographic bone loss and complexity factors. The extent of periodontal disease with respect to stages is shown in Table 3.

### Grading

Of the total study population, 220 participants (48.5%) were assigned to grade B and 19 participants (4.2%) to grade C when grade was calculated from radiographic bone loss only. No periodontitis cases were assigned to grade A. When grade modifiers were included, the number of participants assigned to grade B decreased to 203 (44.7%) while the number assigned to grade C increased to 36 (7.9%) (Table 3). The distribution of study participants with respect to periodontitis presence, stage, grade, and extent is shown in Table 4.

**Table 4:** Distribution of study participants by periodontitis presence, stage, grade, and extent (N = 454)

	Non-periodontitis cases	Stage I	Stage II	Stage III	Stage IV
	n (%)	n (%)	n (%)	n (%)	n (%)
Total	215 (47.4)	0 (0)	75 (16.5)	149 (32.8)	15 (3.3)
*Grade A*					
Localized	–	0 (0)	0 (0)	0 (0)	0 (0)
Generalized	–	0 (0)	0 (0)	0 (0)	0 (0)
*Grade B*					
Localized	–	0 (0)	5 (1.1)	121 (26.7)	0 (0)
Generalized	–	0 (0)	68 (15.0)	3 (0.7)	6 (1.3)
*Grade C*					
Localized	–	0 (0)	0 (0)	17 (3.8)	0 (0)
Generalized	–	0 (0)	2 (0.4)	8 (1.8)	9 (2.0)

Localized; < 30% of teeth involved. Generalized; ≥ 30% of teeth involved

### Interproximal RBL cutoff and its impact on prevalence

When changing the RBL cutoff level for defining a periodontitis case from > 3 mm to > 2 mm, the prevalence of periodontitis cases increased to 91.9% of the study population. Reducing the cutoff level for periodontitis to > 1 mm RBL, increased the prevalence further to 99.6%. Differences in distribution with respect to stages are shown in Table 5.

**Table 5:** Periodontal stages distributed by different interproximal radiographic bone loss cutoffs (N = 454)

RBL (mm)	Non-periodontitis cases n (%)	Periodontitis cases n (%)	Stage I n (%)	Stage II n (%)	Stage III n (%)	Stage IV n (%)
> 1	2 (0.4)	452 (99.6)	2 (0.4)	243 (53.5)	191 (42.1)	16 (3.5)
> 2	37 (8.1)	417 (91.9)	0 (0)	212 (46.7)	189 (41.6)	16 (3.5)
> 3	215 (47.4)	239 (52.6)	0 (0)	75 (16.5)	149 (32.8)	15 (3.3)

## Discussion

This study provides comprehensive data regarding the prevalence of periodontitis in a population of 65 year-olds living in Oslo. Of the total study population, the majority had one or more PPDs of 4 mm or more, and BoP was present in 10% or more sites in one-fourth of the participants. In addition, approximately half of the participants were defined as periodontitis cases according to the 3 mm RBL cutoff, and almost three fourth of those with periodontitis had unstable, recurrent periodontitis. The present study also showed that the pre-selected radiographic bone level for defining a periodontitis case had large impact on the prevalence estimate.

PPD is an important measurement for evaluating periodontal conditions [19, 24]. In the present study, 72.9% of the participants had at least one site with PPD ≥ 4 mm, and PPD ≥ 6 mm was present in 26.6% of the participants. This is in line with results in the study from Troms county in northern Norway [7]. Holde et al. found that 81% of the participants had PPD ≥ 4 mm and 33% of the participants had PPD ≥ 6 mm in at least one site in the age group 65–75 years [7]. In a Swedish population of 60 year-olds, similar prevalences were found where PPD ≥ 4 mm and ≥ 6 mm was present in 87% and 37% respectively [27]. In cross-sectional studies, where the participants are only examined once, it is not possible to distinguish between pseudo-pockets and pockets caused by periodontitis. However, sites with BoP and PPD > 3 mm indicates a need for improvement of oral hygiene and initiation of preventive measures to prevent progression of periodontal disease.

Prevalence data on periodontitis among elderly varies between studies. In accordance with the present study where more than half of the participants were classified as periodontitis cases, a prevalence of 49.7% has been reported in a study population of 18–75 year-old in northern part of Norway using a case definition based on the 2017 World Workshop on the Classification of Periodontal and Peri-implant Diseases and Conditions by measuring bone loss on radiographs [5]. However, in the oldest age group ranging from 65 to 75 years old the prevalence was 80.6%. Other studies from Norway have reported prevalence of periodontitis ranging from 33 to 98% [6, 7, 28] with increasing prevalence in older age groups. These studies were based on radiographic evaluation combined with PPD or calculated CAL. Compared to other countries, the prevalence in the present study is in line with results from a population study in the U.S. where 60% of the participants aged 65 years or older had periodontitis according to the CDC/AAP case definitions [8, 29], and with results from Spain where 50% in the age group 55 years or older was registered with bone loss > 3 mm, however this was measured using CAL and not RBL [30].

In addition to the fact that half of the participants in the present study were classified as periodontitis cases, more than one-third were registered with unstable periodontitis. This indicates a considerable periodontal treatment need among the young elderly in Oslo. Dental health services are readily accessible in Oslo and periodontal treatment expenses are partly subsidized by the Norwegian welfare system, therefore the prevalence of 65-year-olds with unstable periodontitis is surprisingly high. It might be speculated that poor patient compliance with respect to dental hygiene instructions, infrequent dental visits, or underdiagnosed periodontitis might be possible reasons for the high prevalence. These factors should be investigated in future studies.

In the present study, 16.5% of the participants were assigned to stage II, 32.8% to stage III, and 3.3% to stage IV periodontitis. Stødle et al. found a comparable prevalence of stage III (29.1%) and stage IV (3.6%) among 60–69 year-olds in a population in Trøndelag county [6], however, the prevalence of stage II was 58.8% which is a high proportion compared to the present study. In the study from Trøndelag, the prevalence of periodontitis was assessed based on the consensus report from the 2017 World Workshop on the Classification of Periodontal and Peri-implant Diseases and Conditions, and a RBL cutoff of 1.5 mm was used for all age groups. This might explain the higher prevalence of stage II periodontitis compared to the present study. When lowering the RBL cutoff for identifying a periodontitis case from > 3 mm to > 2 mm or > 1 mm in the present study the prevalence of periodontitis increased remarkably to 91.9% and 99.6%. This indicates that a universal threshold for the diagnosis of periodontitis in epidemiological studies is required. It has previously been reported a large variation in the prevalence of periodontitis categorized by CAL threshold of 3 mm (≈ 95%), 4 mm (≈ 75%), 5 mm (≈ 50%), 6 mm (≈ 30%), and 7 mm (≈ 20%) in a population of 65 year-olds in the U.S. [29]. The challenge regarding the use of different case definitions and periodontal examination protocols has previously been highlighted [16, 31, 32]. The consensus report from 2017 [19] has tried to alleviate this and presents cutoffs with respect to CAL for staging periodontitis cases. Interproximal CAL of ≥ 2 mm or ≥ 3 mm are presented as commonly used cutoffs for identifying a periodontitis patient [19], however, no exact value of CAL is given in the 2017 World Workshop case definition. Physiological bone level has been defined as bone level 1 mm to 3 mm apical to the cemento-enamel junction [24]. Ke et al. compared the consensus report using CAL cutoffs for stage II and III with other previously used periodontal classifications [20]. In addition to affecting the prevalences reported, the new classification showed strong associations with known risk factors such as smoking. However, Ke et al. used CAL only and requested further investigation of the epidemiological utility [20]. As described, the staging and grading procedure requires several parameters which are rare in epidemiological studies, like longitudinal design and radiographic determination of bone levels in addition to several clinical parameters [20]. The present study used radiographic bone levels to determine percent bone loss per age and thereby calculating the indirect evidence of progression as described in the consensus report [19]. However, to determine the direct evidence of progression, longitudinal data would be required, and results from the present study may serve as a baseline for follow-up studies in the same study population.

The availability of CAL instead of RBL in the present study could give a more precise estimate of the prevalence. In the study by Eke et al. CAL was used and bone loss in frontal areas, which obviously are not included on BWs, could be identified [29]. This might explain the higher prevalence of periodontitis with the 3 mm cutoff in the study from the U.S. [29] compared to the present study. As previously described, the use of RBL for defining a periodontitis case might lead to underestimation of mild to moderate periodontitis [25]. However, measurements necessary for calculating CAL in addition to all the other parameters required for staging and grading according to the consensus report are time consuming and was not used in the present study due to time limitation. The current study indicates that the consensus report is poorly applicable in large epidemiological studies including several aspects of oral health, and may be better suited for clinical practice and studies investigating periodontitis only.

Another limitation of the present study was that no dental records regarding participants’ treatment history were available. Teeth were registered as lost due to periodontitis if no other reasons for tooth loss such as caries, endodontic lesions, root canal treatments seemed reasonable, and when the bone level in the remaining dentition suggested loss of periodontal attachment. In addition, in cases where the reason for the bone loss was questionable, the case was discussed until a consensus was made. In uncertain cases, the cause was not assigned to periodontitis, and this may have led to an underestimation of both the prevalence and severity of periodontitis.

The response rate in the OM65 study was 58% [21], leading to a sizable proportion of non-respondents and possibility of selection bias. The selection of individuals from the target population was random, however, several factors may have influenced whether individuals agreed to participate or were reachable by phone. Due to ethical considerations, information regarding reasons for not attending the study was not available. Nevertheless, some individuals unsolicited stated periodontitis as a reason for not participating in the study due to tiring and frequent visits to the dentist, which may indicate that the prevalence might be even higher in the target population. However, a lower prevalence of periodontitis among Caucasians compared to individuals of other ethnicities has been reported [33]. Furthermore, low socioeconomic status has also been reported as a risk indicator for attachment loss and increased probing depth [33]. When comparing background characteristics of individuals in the present study with the general population (based on register data from Statistics Norway), western-born and highly educated individuals were overrepresented compared to the target population, and the prevalence might therefore have been underestimated in the present study. In addition, the proportion of current smokers, which is a well-documented risk factor for periodontitis [34, 35], was lower in the present study population compared to the target population which may also have led to an underestimation of periodontitis prevalence. The proportion of individuals with diabetes was similar in the present study population compared to the target population. However, due to the lack of information regarding diseases control in diabetic individuals (HbA1c) in the present study, diabetes was only treated as a grade modifier for shifting the grade from A to B and not from B to C. This may have led to an underestimation of grade C periodontitis. Due to the high proportion of highly educated individuals, low proportion of smokers, public financial support for periodontal treatment, and readily accessible dental health services, one could expect a low risk for periodontitis. Despite this, we found a high prevalence in the present study population. This indicates that the prevalence might be even higher in the target population of 65-year-olds in Oslo and even higher in areas where dental health services are not that readily accessible.

## Conclusions

Periodontitis was common among 65 year-olds living in Oslo, and the majority of those with the disease had unstable, recurrent periodontitis. This indicates a substantial need for periodontal treatment in this population in the years to come. In addition, this study shows that the choice of radiographic bone loss cutoff for defining a periodontitis case affects the prevalence estimates to a large extent. This study also indicates that the staging and grading procedure in the 2018 EFP/AAP classification has major limitations for epidemiologic studies in its current form.

## Data Availability

The datasets used and/or analyzed during the current study are available from the corresponding author upon reasonable request.
